# A Common Neuronal Ensemble in the Lateral Habenula Regulates Ciprofol Anesthesia in Mice

**DOI:** 10.3390/ph17030363

**Published:** 2024-03-11

**Authors:** Kang Zhou, Lin-Chen Zhang, He Zhu, Bei Wen, Jia-Li Tang, Ping-Chuan Yuan, A-Fang Zhu, Yu-Guang Huang

**Affiliations:** 1 Department of Anesthesiology, Peking Union Medical College Hospital, Chinese Academy of Medical Sciences & Peking Union Medical College, Beijing 100730, China; pumc_zhoukang@student.pumc.edu.cn (K.Z.); zhuhe166959@student.pumc.edu.cn (H.Z.); pumc_wenbei@student.pumc.edu.cn (B.W.); tangjiali@pumch.cn (J.-L.T.); 2 Department of Pharmacology, State Key Laboratory of Medical Neurobiology and MOE Frontiers Center for Brain Science, School of Basic Medical Sciences, Shanghai Medical College of Fudan University, Shanghai 200032, China; 20301050108@fudan.edu.cn (L.-C.Z.); 20180042@wnmc.edu.cn (P.-C.Y.); 3 School of Pharmacy, Wannan Medical College, Wuhu 241000, China

**Keywords:** general anesthesia, loss of consciousness, TRAP, gene ablation, chemogenetic

## Abstract

General anesthetics were first used over 170 years ago; however, the mechanisms of how general anesthetics induce loss of consciousness (LOC) remain unclear. Ciprofol, a novel intravenous anesthetic, has been developed by incorporating cyclopropyl into the chemical structure of propofol. This modification offers the benefits of rapid onset and minimal injection pain. Recent studies have revealed that the glutamatergic neurons of the lateral habenula (LHb) play a crucial role in modulating the LOC induced by propofol and sevoflurane. Nevertheless, the specific involvement of LHb in the anesthetic effects of ciprofol remains uncertain. Here, using targeted recombination in active populations (TRAP) combined with electroencephalogram/electromyography recordings and the righting reflex behavioral test, our study revealed that intravenous infusion of ciprofol for 1 h could lead to the induction of c-Fos expression in the LHb in mice. The combination of TRAP and gene ablation, aimed at selectively ablating ciprofol-activated neurons in the LHb, has been shown to facilitate the emergence of ciprofol anesthesia and decrease the proportion of delta waves during the emergence phase. Chemogenetic inhibition of these neurons produced a comparable effect, whereas chemogenetic activation resulted in the opposite outcome. Chemogenetic activation of ciprofol-activated neurons in the LHb delays the emergence of anesthesia and induces a deep hypnotic state during the emergence phase. Taken together, our findings suggest that LHb ciprofol-activated neurons modulate the state of consciousness and could potentially be targeted to manipulate consciousness during ciprofol anesthesia.

## 1. Introduction

Propofol is currently a widely used intravenous anesthetic in clinical anesthesia. However, propofol has some disadvantages, such as injection pain and a relatively high incidence of hypotension, particularly during anesthesia induction [1]. To minimize the occurrence of these side effects, new varieties of intravenous general anesthetics have been developed. Ciprofol is a new type of intravenous anesthetic recently approved for clinical use in China. It forms a chiral structure by adding a cyclopropyl group to the chemical structure of propofol. This modification increases the affinity of ciprofol for γ-aminobutyric acid-type A (GABA_A_) receptors by approximately five times compared to propofol [2,3]. Several studies have shown that ciprofol can reduce the occurrence of injection pain and hypotension during the induction period associated with propofol [4,5,6].

The neural mechanisms of general anesthesia have been extensively studied in the past decade, and one important hypothesis is the sleep-anesthesia shared neural circuit hypothesis [7,8]. Previous studies have found that many nuclei involved in regulating sleep-wakefulness are implicated in the loss of consciousness (LOC) induced by general anesthetics. The locus coeruleus, basal forebrain, thalamic reticular nucleus, paraventricular thalamic nucleus, medial septum, and lateral habenula (LHb) are involved in regulating the LOC caused by propofol [9,10,11,12,13,14,15]. The LHb, as part of the epithalamus, is mainly composed of glutamatergic neurons [16]. It plays a crucial role in connecting the forebrain and midbrain, as well as regulating the dopaminergic and serotonergic systems [17]. It mainly functions in reward processing, stress adaptation, sleep, and circadian rhythm regulation [16,18]. Gelegen and colleagues reported that sedative doses of propofol increased c-Fos expression in the LHb. Additionally, blocking LHb glutamatergic output significantly reduced propofol-induced loss of righting reflex (LORR) [15]. A recent study suggests that LHb glutamatergic neurons and pathways are essential in modulating isoflurane anesthesia [19]. However, it is still unknown whether the LHb is causally involved in the process of unconsciousness induced by ciprofol.

In this study, targeted recombination in active populations (TRAP), electroencephalogram/electromyography (EEG/EMG) recordings, gene ablation, and chemogenetic manipulation were used to investigate the role of a cluster of neurons in the LHb during ciprofol anesthesia. The results showed that ciprofol anesthesia can induce an increase in c-Fos expression in the LHb. The ablation of LHb ciprofol-activated neurons facilitated the emergence of ciprofol anesthesia, and chemogenetic inhibition of these neurons had a similar effect, while chemogenetic activation resulted in opposite effects. Our findings show that LHb ciprofol-activated neurons play a critical role in modulating the recovery of consciousness (ROC) disappearance caused by ciprofol anesthesia.

## 2. Results

### 2.1. Ciprofol Increases c-Fos Expression in the LHb

According to the recommended dosages of propofol and ciprofol used clinically, the effects of ciprofol at 0.4–0.6 mg/kg are comparable to those of propofol at 1.5–2.5 mg/kg. A previous study has found that intravenous infusion of propofol at 10 mg/kg/min for 5.5 min induced LORR in mice [13]. Based on the potency relationship, we observed the effects of ciprofol at 1 mg/kg/min and 2.5 mg/kg/min on LORR in mice. Ciprofol infused at 1 mg/kg/min and 2.5 mg/kg/min induced LORR in 453 (15) s and 150 (10) s (*p* < 0.001), respectively (Figure S2A). In subsequent experiments, a dosage of 2.5 mg/kg/min was used to induce ciprofol anesthesia in mice.

**Figure 1:**
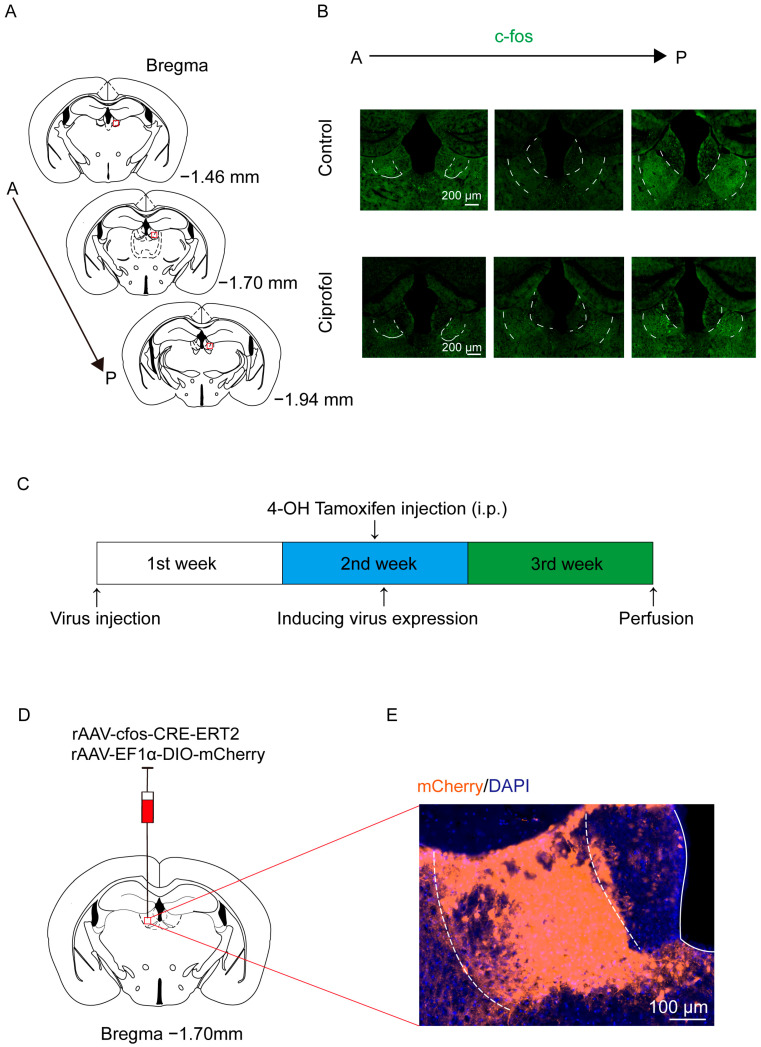
Ciprofol anesthesia increases c-Fos expression in the lateral habenula. (**A**): Schematic diagram of the lateral habenula (LHb) region in the distribution of neurons activated by ciprofol. A, anterior. P, posterior. (**B**): The cluster of cells in the LHb was activated by ciprofol. A, anterior. P, posterior. (**C**): Schematic diagram of TRAP technology to label ciprofol-activated neurons. (**D**): Schematic diagram of virus injection in the LHb. (**E**): Typical diagram of TRAP technology to label ciprofol-activated neurons.

Mice were intravenously infused with ciprofol for 1 h, and an increased number of c-Fos-positive neurons in the LHb per slice was observed (Figure 1A,B). Next, we evaluated the effectiveness of TRAP technology under the conditions of this experiment. We initially injected AAV-cfos-CRE-ERT2 and AAV-EF1α-DIO-mCherry (in a 1:1 uniform mixture) into the LHb region in WT mice, followed by intraperitoneal injection of 4-OH tamoxifen at 1 week after the virus injection (Figure 1C). The mice were then exposed to ciprofol anesthesia for 1 h to label the ciprofol-activated neurons with mCherry. We found that ≈65% of TRAP-labeled neurons were c-Fos positive (Figure S1A,B), suggesting that TRAP is specific for labeling ciprofol-activated neurons (Figure 1E). The above results demonstrate that ciprofol reliably induces the expression of c-Fos in the LHb, and TRAP technology can effectively label these activated neurons.

### 2.2. Ablation of LHb Neurons Facilitates the Emergence of Ciprofol-Induced Anesthesia

**Figure 2:**
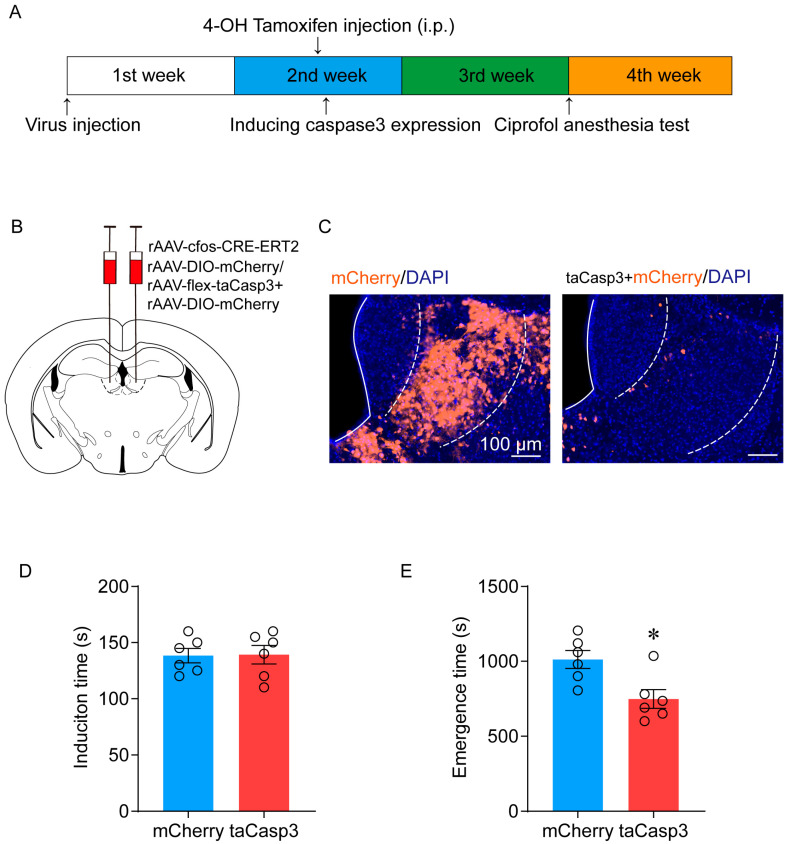
Ablation of LHb neurons facilitates the emergence of ciprofol anesthesia. (**A**): Schematic diagram of TRAP technology to specifically ablate the ciprofol-activated neurons. (**B**): Schematic diagram of virus injection in the LHb. (**C**): Representative immunohistochemical staining. Scale bar, 100 µm. (**D**): Induction time with ciprofol exposure (*n* = 6 for each group). (**E**): Emergence time with ciprofol exposure (*n* = 6 for each group). The asterisk in (**D**,**E**) indicates a significant difference (* *p* < 0.05). Statistical comparisons were conducted using a two-sample independent *t*-test (**D**,**E**). Error bars represent ± SEM.

To investigate a causal relationship, we injected AAV2/9-EF1α-flex-taCasp3 mixed with AAV2/9-EF1α-DIO-mCherry, or a control AAV2/9-EF1α-DIO-mCherry virus, into the LHb to specifically ablate ciprofol-activated neurons using TRAP technology (Figure 2B). In subsequent experiments, as described previously, 4-OH tamoxifen was used to activate the TRAP system. The results showed that compared with the control group, the LHb region of the ablated group exhibited a significant loss of neurons (Figure 2C).

To explore whether ciprofol-activated neurons are potential targets for ciprofol anesthesia, a righting reflex behavioral test was conducted following caspase-3-mediated ablation of ciprofol-activated neurons. Consistently, we observed that ablation of ciprofol-activated cells in the LHb indeed accelerated emergence from anesthesia (from 1012 (60) s to 748 (63) s, *p* = 0.013) (Figure 2E). However, the difference in LORR did not show statistical significance (138 (6) s vs. 139 (8) s, *p* = 0.6) (Figure 2D).

**Figure 3:**
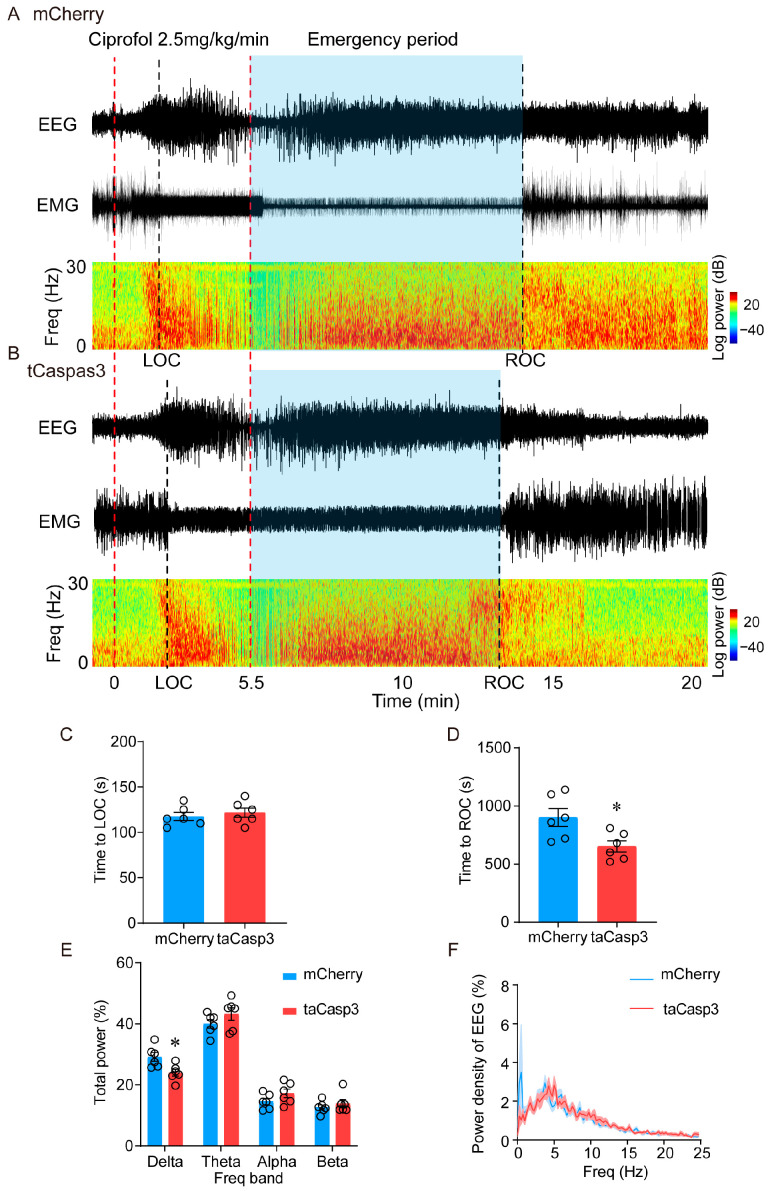
Ablation of LHb neurons decreased the proportion of delta waves during the emergence period. (**A**,**B**): Representative EMG/EEG traces and EEG power spectrum of lesion group and mCherry group, ciprofol at 2.5 mg/kg/min was infused for 5.5 min. (**C**): Time to LOC with ciprofol exposure. (**D**): Time to ROC with ciprofol exposure. (**E**): Relative EEG power of lesion group and mCherry group. (**F**): Normalized power densities of EEG signals of the lesion group and mCherry group. The asterisk in (**D**,**E**) indicates a significant difference (* *p* < 0.05). Statistical comparisons were conducted using a two-sample independent *t*-test (**C**,**D**) and two-way repeated-measures ANOVA followed by Sidak’s post hoc test (**E**). Error bars represent ± SEM. LOC, loss of consciousness, ROC, recovery of consciousness.

Meanwhile, polysomnographic recording (EEG and EMG) was used to assess the depth of anesthesia. Research has indicated that EEG delta (0.5–4 Hz) power gradually increases from the state of awakening to anesthesia [20]. The results showed a significant reduction in the time to ROC in the caspase-3-mediated ablation group of mice, from 690 (72) s to 520 (48) s (*p* = 0.02) (Figure 3A,B,D). This was accompanied by a decrease in the proportion of delta waves during the emergence period, from 29.2 (1.5) % to 24.0 (1.1) % (*p* = 0.039) (Figure 3A,B,E,F). However, there was no significant difference in the EEG spectrum of the induction stage and time to LOC (Figure S2B,C). These findings demonstrate that the ciprofol-activated neurons in the LHb are an important factor in the ROC loss caused by ciprofol.

### 2.3. Activation of LHb Ciprofol-Activated Neurons Delays the Emergence of Anesthesia

**Figure 4:**
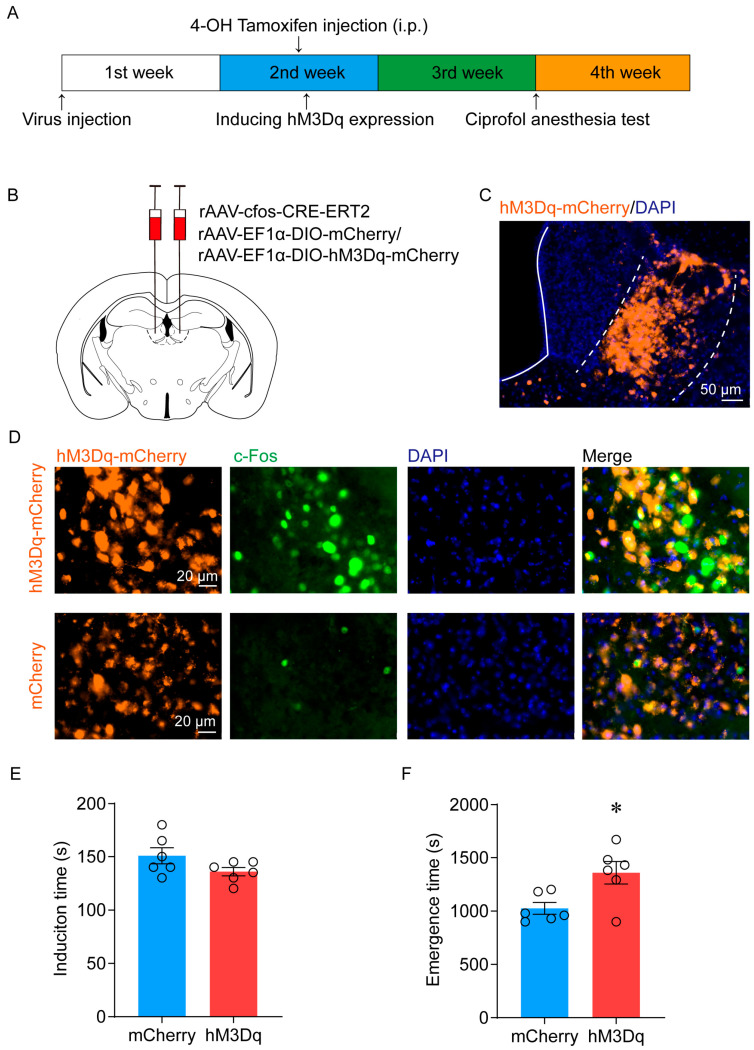
Activation of LHb ciprofol-activated neurons delays the emergence of anesthesia. (**A**): Schematic diagram of TRAP technology to specifically manipulate the ciprofol-activated neurons. (**B**): Schematic diagram of AAV-cfos-CRE-ERT2 and AAV-EF1α-DIO-hM3Dq-mCherry/AAV-EF1α-DIO-mCherry injection in the LHb. (**C**): Representative immunohistochemical staining. Scale bar, 50 µm. (**D**): Representative images of hM3Dq-mCherry/c-Fos/DAPI immunofluorescence in LHb neurons after CNO treatment; scale bar, 20 μm. (**E**): Induction time with ciprofol exposure after intraperitoneal (i.p.) injections of CNO (1 mg/kg) for 1 h (*n* = 6 for each group). (**F**): Emergence time with ciprofol exposure after i.p. injections of CNO (1 mg/kg) for 1 h (*n* = 6 for each group). The asterisk in (**E**,**F**) indicates a significant difference (* *p* < 0.05). Statistical comparisons were conducted using a two-sample independent *t*-test (**E**,**F**). Error bars represent ± SEM. CNO, clozapine-N-oxide.

To further investigate the causal role of LHb ciprofol-activated neurons in ciprofol anesthesia, we injected AAV-cfos-CRE-ERT2 mixed with the Cre-dependent AAV encoding the hM3Dq receptor (AAV-EF1α-DIO-hM3Dq-mCherry) or mCherry (AAV-EF1α-DIO-mCherry) control into the LHb (Figure 4A,B). The typical diagram of hM3Dq-mCherry infection is shown in Figure 4C. There was a notable increase in c-Fos expression in the ciprofol-activated LHb region after clozapine-N-oxide (CNO) administration (1 mg/kg) (Figure 4D).

**Figure 5:**
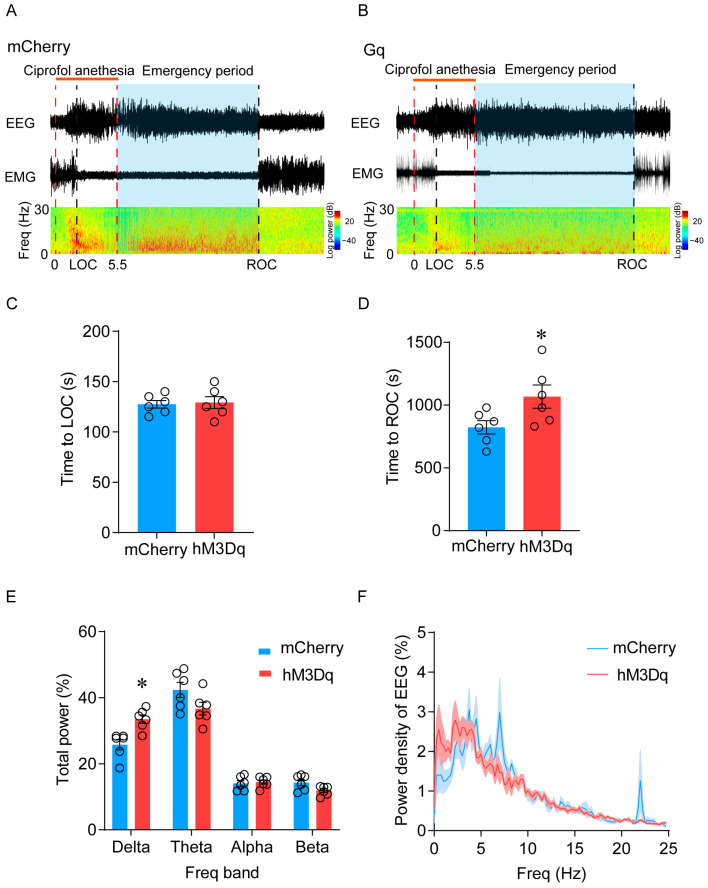
Activation of LHb ciprofol-activated neurons induced a deep hypnotic state during the emergence period. (**A**,**B**): Representative EMG/EEG traces and EEG power spectrum of hM3Dq-mCherry group and mCherry group, ciprofol at 2.5 mg/kg/min was infused for 5.5 min. (**C**): Time to LOC with ciprofol exposure. (**D**): Time to ROC with ciprofol exposure. (**E**): Relative EEG power of hM3Dq-mCherry group and mCherry group. (**F**): Normalized power densities of EEG signals of hM3Dq-mCherry group and mCherry group. The asterisk in (**D**,**E**) indicates a significant difference (* *p* < 0.05). Statistical comparisons were conducted using a two-sample independent *t*-test (**C**,**D**) and two-way repeated-measures ANOVA followed by Sidak’s post hoc test (**E**,**F**). Error bars represent ± SEM. CNO, clozapine-N-oxide, LOC, loss of consciousness, ROC, recovery of consciousness.

Anesthetic behavior tests were performed 1 h after intraperitoneal injection of CNO. Compared with the mCherry group, chemical genetic activation significantly delayed the emergence of mice (from 1025 (53.4) s to 1358 (105.2) s, *p* = 0.02) (Figure 4F). However, there was no significant change in anesthesia induction time (150.8 (7.6) s vs. 135.8 (4) s, *p* = 0.18) (Figure 4E). The onset time of LOC was similar between the hM3Dq-expressing and mCherry control mice (127.5 (3.8) s vs. 129.2 (5.8) s, *p* = 0.82) (Figure 5A–C). In contrast, the time of ROC was significantly prolonged (823.3 (53) s vs. 1068 (92.3) s, *p* = 0.04) (Figure 5A,B,D). In addition, spectral analysis of EEG data revealed that activation of LHb ciprofol-activated neurons induced a significant increase in delta power in the hM3Dq group during the emergency period (25.8 (1.6) % vs. 33.5 (1.3) %, *p* = 0.015) (Figure 5A,B,E,F), which was consistent with the phenomenon of delayed emergency. In addition, there were no differences in the EEG spectrum during the induction period (Figure S3A,B). In summary, chemogenetic activation of LHb ciprofol-activated neurons delayed the emergence from ciprofol anesthesia and induced a deep hypnotic state during the emergence period.

### 2.4. Inhibition of LHb Ciprofol-Activated Neurons Facilitates the Emergence of Anesthesia

**Figure 6:**
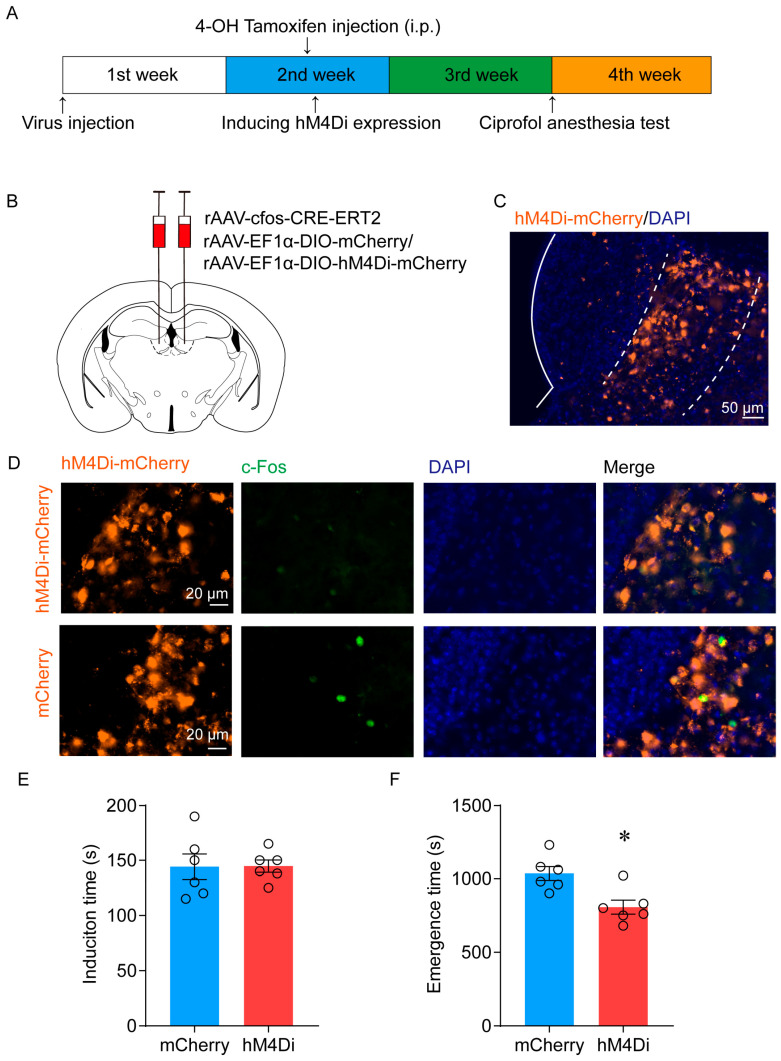
Inhibition of LHb ciprofol-activated neurons facilitates the emergence of anesthesia. (**A**): Schematic diagram of TRAP technology to specifically inhibit the ciprofol-activated neurons. (**B**): Schematic diagram of AAV-cfos-CRE-ERT2 and AAV-EF1α-DIO-hM4Di-mCherry/AAV-EF1α-DIO-mCherry injection in the LHb. (**C**): Representative immunohistochemical staining. Scale bar, 50 μm. (**D**): Representative images of hM3Di-mCherry/c-Fos/DAPI immunofluorescence in LHb neurons after CNO treatment; scale bar, 20 μm. (**E**): Induction time with ciprofol exposure after intraperitoneal (i.p.) injections of CNO (1 mg/kg) for 1 h (*n* = 6 for each group). (**F**): Emergence time with ciprofol exposure after i.p. injections of CNO (1 mg/kg) for 1 h (*n* = 6 for each group). The asterisk in (**E**,**F**) indicates a significant difference (* *p* < 0.05). Statistical comparisons were conducted using a two-sample independent *t*-test (**E**,**F**). Error bars represent ± SEM. CNO, clozapine-N-oxide.

To investigate the essential role of LHb ciprofol-activated neurons in ciprofol anesthesia, we utilized the inhibitory hM4Di receptor (AAV-EF1α-DIO-hM4Di-mCherry) or mCherry (AAV-EF1α-DIO-mCherry) to specifically inhibit ciprofol-activated neurons in the LHb through TRAP technology (Figure 6A,B). The typical diagram of hM4Di-mCherry infection is shown in Figure 6C. c-Fos staining was used to confirm the effectiveness of virus expression, and the findings indicated minimal c-Fos expression in the ciprofol-activated LHb region following CNO administration (1 mg/kg) (Figure 6D).

**Figure 7:**
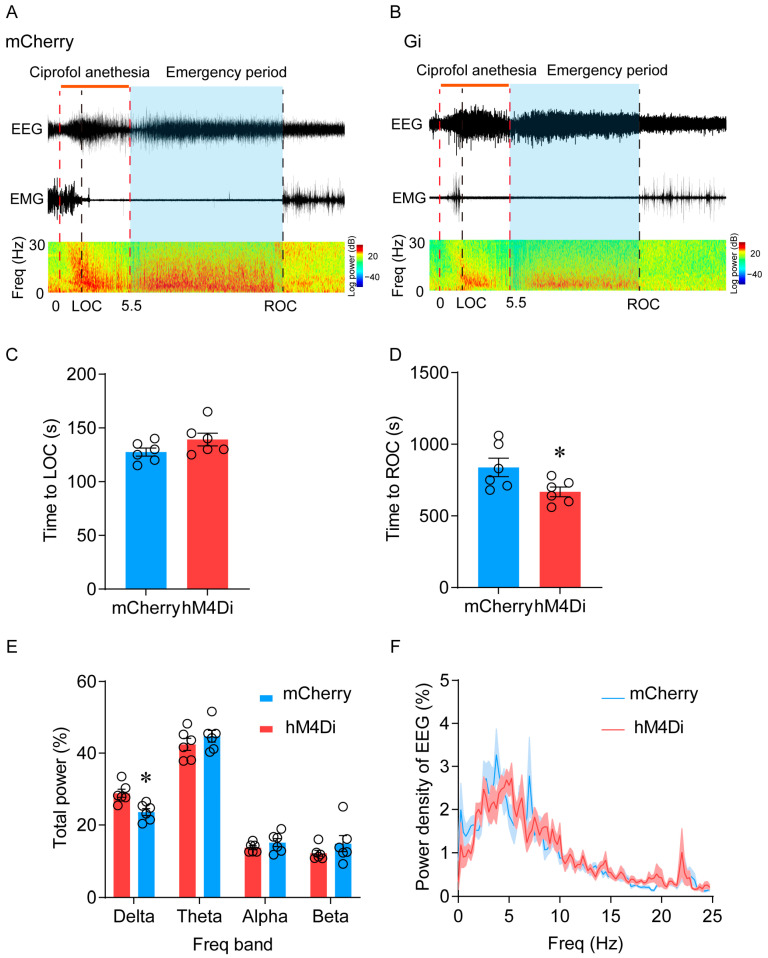
Inhibition of LHb ciprofol-activated neurons decreased the proportion of delta waves during the emergence period. (**A**,**B**): Representative EMG/EEG traces and EEG power spectrum of hM4Di-mCherry group and mCherry group, ciprofol at 2.5 mg/kg/min was infused for 5.5 min. (**C**): Time to LOC with ciprofol exposure. (**D**): Time to ROC with ciprofol exposure. (**E**): Relative EEG power of hM4Di-mCherry group and mCherry group. (**F**): Normalized power densities of EEG signals of hM3Dq-mCherry group and mCherry group. The asterisk in (**D**,**E**) indicates a significant difference (* *p* < 0.05). Statistical comparisons were conducted using a two-sample independent *t*-test (**C**,**D**) and two-way repeated-measures ANOVA followed by Sidak’s post hoc test (**E**). Error bars represent ± SEM. CNO, clozapine-N-oxide, LOC, loss of consciousness, ROC, recovery of consciousness.

Anesthetic behavior tests were performed 1 h after intraperitoneal injection of CNO. There is no significant difference in the induction time between the two groups (from 144.2 (11.6) s to 144.7 (5.5) s; *p* = 0.97) (Figure 6E). In the hM4Di-CNO group, the emergence time was reduced from 1035 (47.5) s to 806.7 (47.5) s (*p* = 0.007) compared with the mCherry-CNO group (Figure 6F). Additionally, the interval of time to ROC in the hM4Di-CNO group was shortened from 838.3 (64.5) s to 668.3 (34) s compared to the control group (*p* = 0.04) (Figure 7D). EEG recordings revealed a decrease in the proportion of delta waves in the hM4Di-expressing mice during the emergence period (from 28.8 (1.1) % to 23.6 (1.0) %, *p* = 0.023) (Figure 7A,B,E,F). This suggests that the selective inhibition of LHb ciprofol-activated neurons resulted in a lighter state of anesthesia during the emergence of ciprofol anesthesia. These results demonstrate the essential role of LHb ciprofol-activated neurons in ciprofol anesthesia.

## 3. Discussion

In this study, we manipulated ciprofol-activated neurons in the LHb to illuminate the role of LHb in ciprofol-induced anesthesia using genetic ablation and chemogenetics. The results revealed that a group of neurons was activated by ciprofol in the LHb. Specific lesions of these neurons and chemogenetic inhibition of ciprofol-activated neurons resulted in a faster emergence from ciprofol anesthesia. In contrast, chemogenetic activation of ensembles delayed the onset of anesthesia.

Ciprofol, a novel intravenous general anesthetic, has been proven to have significant sedative effects in several clinical trials. It is widely used for inducing clinical anesthesia in the operating room and for sedation during diagnostic and therapeutic procedures for patient comfort. Compared to propofol, ciprofol anesthesia has the advantages of lower incidence of induced hypotension and milder injection pain [6,21,22]. However, there is currently almost no research on the neural circuit mechanism of ciprofol. The lateral habenula, as a component of the diencephalon, has been shown to play an important role in regulating REM sleep and consciousness loss caused by propofol and isoflurane anesthesia in previous studies [15,19,23]. The results of the study showed that the anesthesia dose of ciprofol induced a significant increase in c-Fos expression in the LHb, which is similar to the effects of propofol and isoflurane. A previous study showed that sedative doses of propofol induced an increase in c-Fos expression in the LHb [15], while another study confirmed that isoflurane increases glutamate neuron activity in the LHb using calcium fiber photometry recording [19].

In the study, specific ablation of ciprofol-activated cells in the LHb hastened emergence from anesthesia and reduced the proportion of delta waves during the recovery period. However, gene ablation of these neurons had no significant effect on the induction of ciprofol anesthesia. Previous studies, which primarily focused on the glutamatergic neurons in the LHb, have shown a different finding from our study [15,19]. The previous results showed that blocking glutamatergic output from the LHb greatly diminished the sedative effects of propofol. The mice in the lesion group had almost no difficulty in achieving a sedated state, with the disappearance of the righting reflex. In a subsequent study, Liu and colleagues found that lesioning glutamatergic neurons in the LHb decreased the sensitivity of mice to sevoflurane, resulting in prolonged LORR time and shortened recovery of the righting reflex (RORR) time [19]. It is worth noting that the subtypes of neurons in the LHb are relatively diverse [16,24,25], with a predominance of glutamatergic neurons [26]. In this study, we utilized TRAP technology in combination with gene ablation to precisely ablate neurons activated by ciprofol to explore the role of LHb in ciprofol anesthesia. We found that ablating neurons activated by ciprofol accelerated the emergency of anesthesia, which is consistent with the results of the two studies mentioned above [15,19]. However, no effect of ablation on anesthesia induction was found in this study. The lack of impact on anesthesia induction by the lesion may be attributed to the fact that the ablated neurons were only a subset of the neurons in the LHb. As a result, the effect of ablationon anesthesia may be less significant compared to the previous studies.

In the chemogenetic activation experiment, we observed that systemic injection of CNO to activate a specific group of neurons did not significantly affect anesthesia induction. However, mice in the hM3Dq group maintained a deep hypnotic state during the emergence period. Previous studies have found that chemogenetic activation was able to increase the sensitivity of mice to propofol and sevoflurane and shorten induction time [15,19]. However, this phenotype was not found in our study. This may be explained by the following aspects. The LHb was preferentially activated during the ciprofol anesthesia in mice. These increases may have a “ceiling effect” on further chemogenetic activation of ciprofol-activated neurons in anesthetized mice. This effect could explain why chemogenetic activation of this group of neurons only influenced emergence. Xu and his colleagues demonstrated similar results in their study [27]. In addition, in the series of studies by Dong et al. [28,29,30], it was found that some nuclei only participated in anesthesia emergency and did not participate in anesthesia induction, suggesting that general anesthesia emergency may not be a passive process after drug metabolism, but rather some neurons actively participate in emergency.

The LHb, serving as an excitatory hub, receives inputs from diverse limbic forebrain and basal ganglia structures and targets several important midbrain structures, including the rostromedial tegmental nucleus (RMTg), ventral tegmental area (VTA), and dorsal and median raphe nuclei (DRN/MRN) [16,25,31,32]. The GABAergic neurons of the RMTg have been shown to play an important role in regulating NREM and REM sleep [33,34,35]. From the perspective of the shared neural mechanism of sleep and general anesthesia, the projection of the LHb to RMTg may be one of the circuits regulating the recovery from ciprofol anesthesia. Liu and colleagues found that optogenetic activation of the synaptic terminals of LHb glutamatergic neurons in the RMTg produced a hypnosis-promoting effect during isoflurane anesthesia [19]. This provides some evidence for the neural mechanism. Furthermore, selectively activating dopamine neurons in the VTA induced sustained wakefulness, as described in a previous study [36]. Wallace and colleagues identified four neuronal subtypes that selectively targeted dopaminergic and GABAergic cells in the VTA [25]. The indirect inhibition of dopamine neurons in the VTA by the LHb through GABAergic neurons may be one of the mechanisms influencing anesthesia emergence, as mentioned in previous research [15]. In addition, previous studies have shown that the LHb is extensively connected to the DRN through a complex network of parallel, topographically organized direct and indirect pathways [37,38]. The LHb can directly project to the DRN or indirectly innervate the DRN through GABAergic neurons of RMTg [37], with indirect innervation playing a dominant role. Based on previous research into the involvement of the DRN in the recovery from general anesthesia [28,39,40], we speculate that the LHb may indirectly modulate the function of the DRN in ciprofol anesthesia via the GABAergic neurons of RMTg.

This study has several limitations. As mentioned above, we did not further explore the neural circuit mechanisms underlying the role of LHb in ciprofol anesthesia. Second, certain methods for recording neuronal activities in vivo have not been utilized to further confirm the role of LHb during ciprofol anesthesia. We have not yet confirmed the types of neurons activated by ciprofol, which may require further research to explore the subtypes of these neurons. Fluorescence in situ hybridization and RNA sequencing are helpful for confirming the types of neurons activated by general anesthetics in the LHb. Fiber optic calcium signaling and two-photon calcium imaging are helpful for exploring the real-time changes in activated neurons in the LHb during anesthesia. Although the righting reflex has been widely used in the study of anesthesia behavior, this evaluation method has a certain degree of subjectivity, which may have some impact on the experimental results.

In general, the study identified a specific group of neurons that are activated by ciprofol. Inhibiting or lesioning these neurons in mice facilitates the emergence of ciprofol anesthesia while activating them prolongs the recovery time. This finding suggests that the LHb could be a potential target for ciprofol-induced anesthesia.

## 4. Materials and Methods

### 4.1. Mice

Adult C57BL/6J male mice were provided by the Laboratory Animal Center at Fudan University. Wild-type mice (C57BL/6) were group-housed in a stable temperature (22–24 °C) and humidity (45–55%) animal house with a 12-h (7:00 to 19:00) light/dark cycle [41]. All experiments were conducted using adult male mice aged 8–12 weeks, weighing approximately 25–28 g. The mice were randomly assigned to the experimental groups after obtaining approval from the Animal Ethics Committee (20190221-059). All procedures followed the guidelines of the NIH (United States) for the care and use of animals. All surgical procedures were performed under general anesthesia using isoflurane. Analgesics were administered postoperatively to reduce postoperative pain, and the mice were placed on a thermal blanket for insulation. The number of mice used during the experiment was minimized as much as possible.

### 4.2. Stereotaxic Surgery

Animals were anesthetized with 2% isoflurane and maintained at 1–1.5% isoflurane during the surgery. The animals were then placed in a stereotaxic frame (RWD Life Science, Shenzhen, China). The standard surgical procedure was carried out to expose the brain surface above the LHb. Small craniotomy burr holes were made, and 140 nL of virus (AAV-cfos-CRE-ERT2 mixed with AAV2/9-EF1α-flex-taCasp3/AAV-DIO-hM3Dq/AAV-DIO-hM4Di/AAV-DIO-mCherry, 1:1) (BrainVTA, Wuhan, China) was injected into bilateral LHb (anteroposterior [AP] = −1.4 mm, mediolateral [ML] = ±0.44 mm, dorsoventral [DV] = −2.95 mm). The virus injection was administered over a 10-min period using the micro-injection pump (R480, RWD Life Science, Shenzhen, China). For EEG/EMG electrode implantation, two screws were inserted into the skull on top of the right cortex at AP −3.50 mm, ML 3.00 mm, and AP 1.50 mm, ML 1.50 mm. Two EMG electrodes were placed into the dorsal neck musculature, as described previously [42,43]. After surgery, the mice were placed on a heating pad for postoperative recovery. Mice were housed for 1-2 weeks for complete recovery before experiments.

### 4.3. Labeling-Activated Ensembles

TRAP was used to label ciprofol-activated neurons through the cfos-CRE-ERT2 system [44,45]. AAV-cfos-CRE-ERT2 and Cre-dependent AAV encoding the caspase-3/hM3Dq/hM4Di receptor were first injected (1:1 uniform mixing) into the LHb region in WT mice, followed by intraperitoneal injection of 4-OH tamoxifen (H6278, Sigma-Aldrich, Merck KGaA, Darmstadt, Germany) prepared in a mixed oil solution (of nine parts corn oil and 1 part ethanol) at 1 week after the virus injection (50 mg/kg). These mice were then exposed to ciprofol anesthesia for 1 h to label the ciprofol-activated neurons.

### 4.4. Ciprofol Anesthesia in Mice

Continuous stable tail vein propofol infusion was achieved as described in a previous study [13]. The same method was used for the tail vein injection of ciprofol. Every mouse was put into the cage, and a needle with a #4.5 size, connected to a clear tube with an inner diameter of 0.45 mm, was inserted into the tail vein. The needle was securely attached to the tail using cyanoacrylate glue, and the tube was connected to the micro-injection pump (Syringe Pump 22; Harvard Apparatus, Boston, MA, USA). Ciprofol at 2.5 mg/kg/min was infused via the caudal vein for 5.5 min to induce LOC and LORR. Then, ciprofol at 0.8 mg/kg/min was infused continuously to maintain LOC and LORR. In the righting reflex behavioral test, ciprofol anesthesia was maintained for 30 min. In the EEG-recorded experiments, only the induced dose of ciprofol was used.

### 4.5. Behavioral Test

After the mice started receiving ciprofol infusion, the infusion cage was regularly rotated to flip the mouse onto its back every 15 s to test its ability to right itself. If a mouse failed to turn over onto its four paws within 30 s, it was in a state of LORR. The RORR was determined by the time it took for the mouse to return to its normal position with all four feet on the ground. The induction time was defined as the period between the start of ciprofol infusion and the occurrence of LORR. The emergence time was defined as the duration from the cessation of ciprofol infusion to the RORR. LOC was determined as the moment when the EMG activity ceased, and the mice were in the lateral position, in conjunction with the EEG pattern changes from low amplitude voltage fast waves to high amplitude voltage slow waves. The onset of ROC was identified as the point when sustained EMG activity (>20 s) and low amplitude voltage fast EEG waves first appeared [13,20,43].

For selective ablation of ciprofol-activated neurons in the LHb, AAV2/9- EF1α-DIO-caspase-3 mixed with AAV2/9-EF1α-DIO-mCherry, or control AAV2/9-EF1α-DIO-mCherry virus was injected into the LHb to specifically ablate ciprofol-activated neurons through TRAP technology. For chemogenetic manipulations, the mice in the mCherry control, hM3Dq-mCherry, and hM4Di-mCherry groups were injected with CNO (1 mg/kg) (C4759, LKT, USA) 1 h before the behavioral test and EEG recording. There was at least a 5-day interval between CNO administrations in the same mouse. A detailed experimental protocol can be found in the Supplementary Materials. The experimental groups are blinded to the experimenters’ testing behavior.

### 4.6. EEG/EMG Recording and Analysis

EEG/EMG signals were amplified and filtered (Biotex, Kyoto, Japan; 0.5–30 Hz for EEG, 20–200 Hz for EMG), then digitized (Biotex, Kyoto, Japan), and recorded by VitalRecorder software 1.12.10 (KISSEI COMTEC, Nagano, Japan) [46,47]. The raw EEG data were calculated using Fast Fourier transformation with SleepSign software 3.0 to analyze the EEG spectrum in experiments. EEG power spectra were computed for several epochs (5 s per epoch) in each condition. The mean spectral density of all the epochs in each condition was sorted into successive 0.25 Hz bins, and the absolute EEG power of each 0.25 Hz bin was stratified into 0.5–4 Hz, 4–10 Hz, 10–15 Hz, and 15–25 Hz corresponding to delta, theta, alpha, and beta powers, respectively [20,43].

### 4.7. Immunohistochemistry

Immunofluorescence was performed as described previously [20,48]. The mice were deeply anesthetized with pentobarbital sodium and intracardially perfused with phosphate-buffered saline (PBS), followed by 4% paraformaldehyde (PFA) in PBS. For chemogenetic groups, mice were intraperitoneal injection of CNO 1 h before anesthesia. The mouse brain was removed and fixed for 6 h with 4% PFA at 4 °C after perfusion. For dehydration, brain samples were placed in 30% sucrose diluted with PBS until they sank to the bottom at 4 °C. After dehydration, brain samples were coronally sectioned into 30-µm brain slices via a freezing microtome (CM1950; Leica, Wetzlar, Germany) and collected in PBS. The c-Fos staining procedure was conducted as follows: Brain slices were initially washed three times with PBS for 5 min each time, followed by a 30 min incubation in 0.3% Triton-100x (RH30056, Bio Ruler, Danbury, CT, USA). Subsequently, the brain slices were blocked with 5% BSA (RH33151, Bio Ruler, Danbury, CT, USA) for 1 h and then incubated with the primary antibody (1:3000, ABE457, Millipore, Boston, MA, USA) at 4 °C for 24 h. Afterward, the brain slices were washed three times with PBS and incubated with a donkey-anti-rabbit Alexa488 secondary antibody (1:1000, 711-545-152, Jackson ImmunoResearch, Chester, PA, USA) at room temperature for 2 h. Following another three washes with PBS, the brain slices were mounted on glass slides and coverslipped using DAPI Fluormount-G (Cat# 0100-20, SouthernBiotech, Birmingham, AL, USA). Fluorescent images were captured using an Olympus fluorescence microscope (VS120, Olympus, Tokyo, Japan). The brain tissues from all experimental animals were sectioned for verification, and only data from mice with confirmed virus infection were included for analysis.

### 4.8. Data Analysis and Statistics

All data are expressed in the form of mean ± standard error (mean ± SEM). All data were tested for normality using Shapiro–Wilk normality tests before analysis. If the data passed the normality test, the parametric test was used; otherwise, the non-parametric test was employed. The comparison between two groups of normally distributed data used a two-sample independent *t*-test. One-way or two-way repeated-measures ANOVAs followed by Tukey or Sidak’s post hoc comparison tests were used for multiple comparisons. If the data were not normally distributed, Wilcoxon signed-rank tests or Mann–Whitney U tests were used for further analysis. GraphPad Prism 9.0 (GraphPad Software, San Diego, CA, USA) and SPSS 23.0 software (IBM, Armonk, NY, USA) were used for statistical analyses. In all cases, *p* < 0.05 was considered statistically significant.

## 5. Conclusions

The study identified a common neuronal ensemble in the LHb that regulates ciprofol anesthesia in mice. Inhibiting or lesioning these neurons in mice facilitates the emergence of ciprofol anesthesia while activating them prolongs the recovery time.

## Data Availability

The data presented in this study are available on request from the corresponding author.

## Supplementary Materials

The following supporting information can be downloaded at https://www.mdpi.com/article/10.3390/ph17030363/s1, Figure S1A: Representative images of mCherry/c-Fos/DAPI immunofluorescence in LHb neurons after ciprofol infusion; scale bar, 50 μm. Figure S1B: Histograms showing the specificity of TRAP technology, related to Figure1E. Figure S2A: Effects of ciprofol at 1 mg/kg/min and 2.5 mg/kg/min on LORR in mice. Figure S2B: Relative EEG power of lesion group and mCherry group during the induction period. Figure S2C: Normalized power densities of EEG signals of lesion group and mCherry group. Figure S3A: Relative EEG power of the hM3Dq-mCherry group and mCherry group during the induction period. Figure S3B: Normalized power densities of EEG signals of hM3Dq-mCherry group and mCherry group. Figure S3C: Relative EEG power of the hM4Di-mCherry group and mCherry group during the induction period. Figure S3D: Normalized power densities of EEG signals of hM4Di-mCherry group and mCherry group. Figure S4: Schematic diagram of the main findings of the study.
