# Supplementary material for: A Common Neuronal Ensemble in the Lateral Habenula Regulates Ciprofol Anesthesia in Mice
Source: Pharmaceuticals (Basel). 2024 Mar 11;17(3):363. doi: 10.3390/ph17030363 (PMC10975929; doi:10.3390/ph17030363)
Supplement: Supplementary file 1 [file pharmaceuticals-17-00363-s001.zip › pharmaceuticals-2891928-supplementary.pdf]

Supplemental Information

A common neuronal ensemble in the lateral habenula regulates ciprofol anesthesia in mice

Kang Zhou, Lin-Chen Zhang, He Zhu, Bei Wen, Jia-Li Tang, Ping-Chuan Yuan, A-Fang Zhu, Yu-Guang Huang

Supplementary material

Materials and Methods

Genetic ablation

For the selective ablation of ciprofol-activated neurons in the LHb, AAV2/9-EF1α-DIO-caspase-3 mixed with AAV2/9-EF1α-DIO-mCherry (lesion group), or control AAV2/9-EF1α-DIO-mCherry virus (control group), was injected into the LHb to specifically ablate ciprofol-activated neurons through TRAP technology. One week after the micro-injection, we induced caspase-3 expression by intraperitoneal injection of 4-OH tamoxifen and tail vein injection of ciprofol. Three weeks post-injection, assessments were conducted to evaluate the induction time, emergence time, and EEG/EMG recordings associated with ciprofol anesthesia in both experimental groups. The specifics of the behavioral experiments are outlined in the section titled "Chemogenetic Manipulations." Final histological analyses were conducted approximately six weeks after the AAV injection, upon the completion of all behavioral experiments.

Chemogenetic manipulations

For chemogenetic manipulations, AAV-DIO-hM3Dq/AAV-DIO-hM4Di/AAV-DIO-mCherry were used. One week after the virus injection, we induced hM3Dq/hM4Di expression by intraperitoneal injection of 4-OH tamoxifen and tail vein injection of ciprofol. The mice in the mCherry-control, hM3Dq-mCherry, and hM4Di-mCherry groups were injected with CNO (1 mg/kg) (C4759, LKT, USA) 1 h before the behavioral test and EEG recording. There was a minimum 5-day interval between successive CNO administrations in the same mouse. For the determination of induction time, ciprofol was administered at a rate of 2.5 mg/kg/min. Subsequently, the infusion cage

was gently rotated every 15 seconds to position the mouse on its back, assessing for the righting reflex. The induction time was characterized as the duration from the onset of ciprofol administration to the onset of the Loss of Righting Reflex (LORR). After induction, the mouse was kept under ciprofol anesthesia at a rate of 0.8 mg/kg/min for 30 min and positioned in the supine position with proper temperature control. Emergence time was defined as the period from the cessation of ciprofol administration to the moment the mouse successfully regained an upright posture.

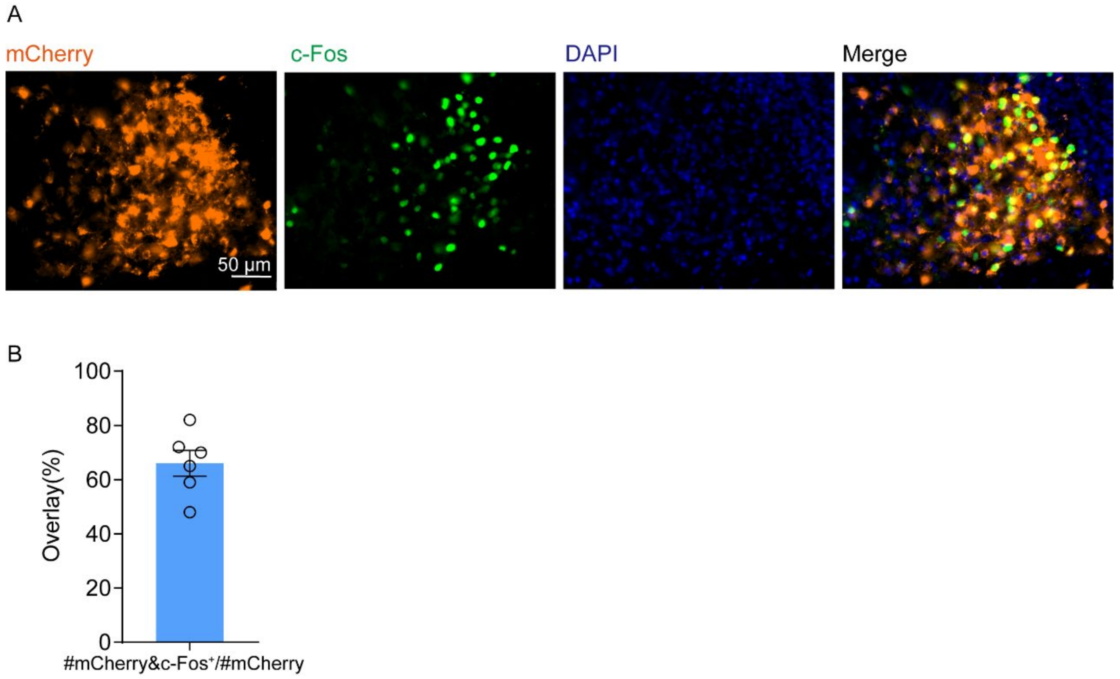

Figure S1A. Representative images of mCherry/c-Fos/DAPI immunofluorescence in LHb neurons after ciprofol infusion; scale bar, 50 μm.

Figure S1B. Histograms showing the specificity of TRAP technology, related to Figure 1E.

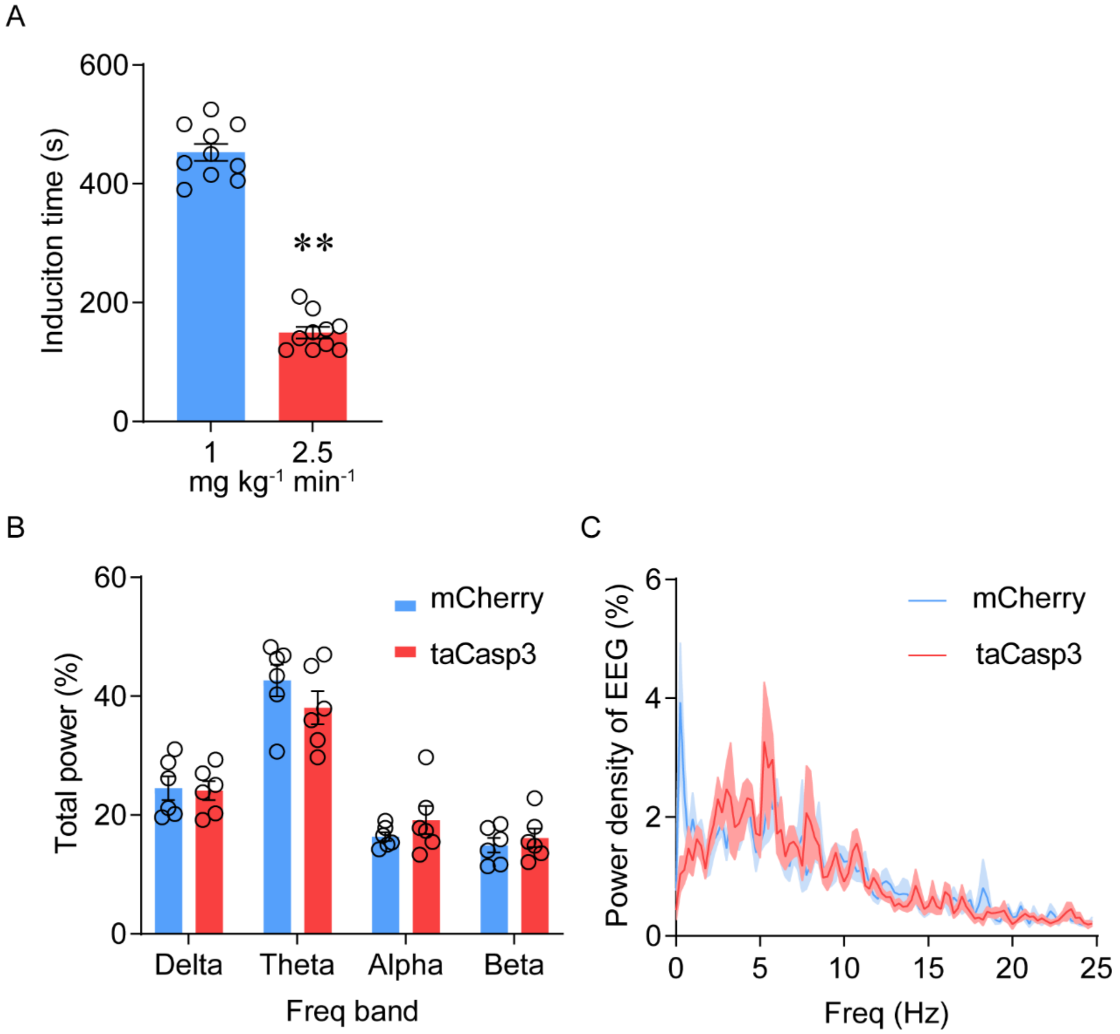

Figure S2A. Effects of ciprofol at 1 mg/kg/min and 2.5 mg/kg/min on LORR in mice.

Figure S2B. Relative EEG power of lesion group and mCherry group during induction period.

Figure S2C. Normalized power densities of EEG signals of lesion group and mCherry group.

The asterisk in (A) indicates a significant difference (***p* < 0.01). Statistical comparisons were conducted using Student's two-tailed paired t test (A) and two-way repeated-measures ANOVA followed by Sidak's post hoc test (B). Error bars represent ± SEM.

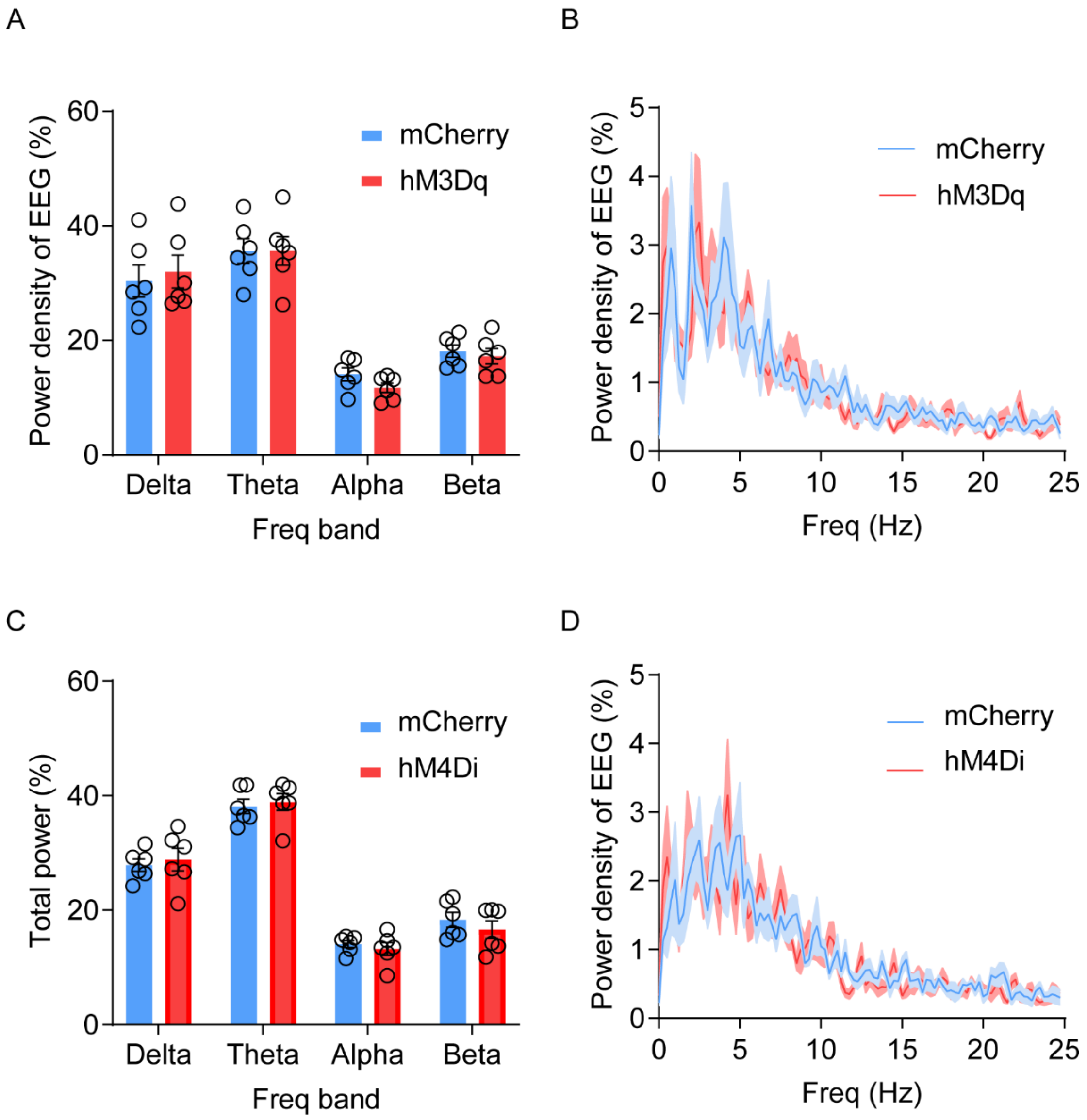

Figure S3A. Relative EEG power of hM3Dq-mCherry group and mCherry group during induction period.

Figure S3B. Normalized power densities of EEG signals of hM3Dq-mCherry group and mCherry group.

Figure S3C. Relative EEG power of hM4Di-mCherry group and mCherry group during induction period.

Figure S3D. Normalized power densities of EEG signals of hM4Di-mCherry group and mCherry group.

Statistical comparisons were conducted using Student's two-tailed paired t test (A,C) and two-way repeated-measures ANOVA followed by Sidak's post hoc test (B). Error bars represent ± SEM.

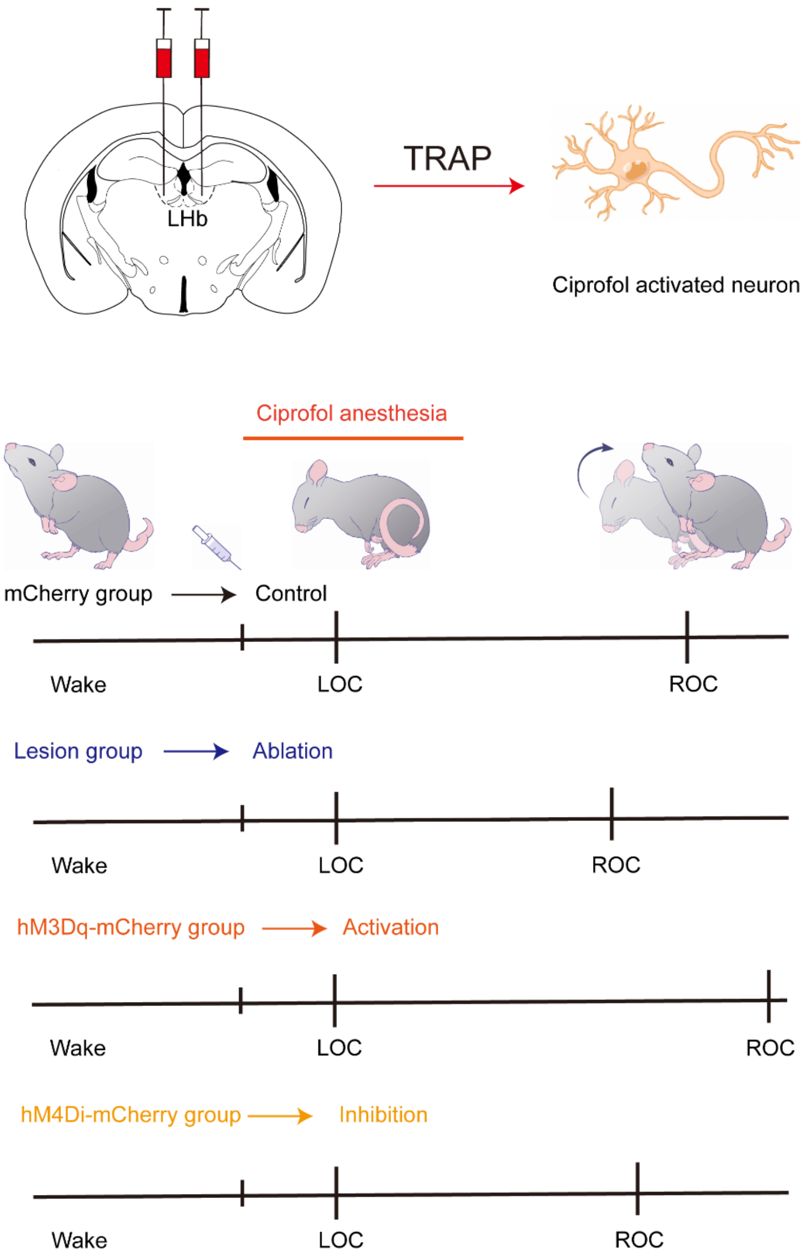

Figure S4. Schematic diagram of the main findings of the study.
